# Supplementary material for: Evaluation of an ultra-portable X-ray system with automated interpretation for tuberculosis active case finding in carceral settings: a diagnostic test accuracy study
Source: BMC Infect Dis. 2025 Nov 3;25:1480. doi: 10.1186/s12879-025-11835-0 (PMC12581292; doi:10.1186/s12879-025-11835-0)
Supplement: Supplementary file 1 — Supplementary Material 1 [file 12879_2025_11835_MOESM1_ESM.pdf]

Identification

Record ID

____

Date of signing of the TCLE

____

Time of signing the TCLE

____

RGI

____

RGI (confirmation)

____

Check the RGI entered.

Name

____
(Full name)

Social name

____
(Full name, if applicable)

Date of birth

____

CPF

____
(Numbers only, 11 digits)

CNS

____
(Numbers only, 15 digits)

CNS (2)

____
(Numbers only, 15 digits)

CNS (3)

____
(Numbers only, 15 digits)

CNS (4)

____
(Numbers only, 15 digits)

CNS (5)

____
(Numbers only, 15 digits)

Mother's name

____
(Full name)

Race

- ◯ White
- ◯ Black
- ◯ Brown
- ◯ Yellow
- ◯ Indigenous

Date of first arrest

____
(Inmate history)

Date of entry into prison

____

It is condemned

- ◯ Yes
- ◯ No
- ◯ No information

Time of condemnation ______ years ______ months

Time remaining

____
(In months)

Time since first arrest

____
(In months)

Time that is stuck

____
(In months)

Machine Translated by Google

Sociodemographic data

Record ID

____

Name: [common_arm_1][name]

Date of birth: [comum_arm_1][dt_nascimento]

TCLE signing date: [comum_arm_1][dt_tcle]

Date of data collection

____

Time of data collection

____

Age

____

Presidio

- ◯ EPJFC
- ◯ IPCG
- ◯ CPAIG
- ◯ Gameleira I
- ◯ Gameleira II
- ◯ PTRAN
- ◯ Patronage
- ◯ Monitoring

Block (EPJFC)

- ◯ Pavilion 1A
- ◯ Pavilion 1B
- ◯ Pavilion 2A
- ◯ Pavilion 2B
- ◯ Pavilion 3
- ◯ Pavilion 4A
- ◯ Pavilion 4B
- ◯ Pavilion 6 (RDD)

Block (IPCG)

- ◯ 1A - Solarium 1A
- ◯ 1A - Solarium 2A
- ◯ 1A - Solarium 3A
- ◯ 1B - Solarium 1B
- ◯ 1B - Solarium 2B
- ◯ 1B - Solarium 3B
- ◯ 2A - Solarium 1A
- ◯ 2A - Solarium 2A
- ◯ 2A - Solarium 3A
- ◯ 2B - Solarium 1B
- ◯ 2B - Solarium 2B
- ◯ 2B - Solarium 3B
- ◯ 2B - Solarium G
- ◯ 2B - CD

Block (CPAIG)	◯ Pavilion A<br>◯ Pavilion B<br>◯ Pavilion C<br>◯ Pavilion D<br>◯ Pavilion F<br>◯ Disciplinary
Block (Gameleira I)	◯ Pavilion 1<br>◯ Pavilion 2<br>◯ Pavilion 3<br>◯ Pavilion 4<br>◯ Disciplinary<br>◯ Inclusion<br>◯ Work
Block (Gameleira II)	◯ Pavilion 1A<br>◯ Pavilion 1B<br>◯ Pavilion 1D<br>◯ Pavilion 2B<br>◯ Pavilion 2C<br>◯ 2D Pavilion<br>◯ Pavilion 3A<br>◯ Pavilion 3B<br>◯ 3D Pavilion<br>◯ Pavilion 3E<br>◯ Pavilion 3F<br>◯ Pavilion 4<br>◯ Inclusion A<br>◯ Inclusion B<br>◯ Health
Block (PTRAN)	◯ Pavilion 1A<br>◯ Pavilion 1B<br>◯ Pavilion 2A<br>◯ Pavilion 2B<br>◯ Pavilion 2B - Disciplinary
What is your cell number?	________________________________
Height	________________________________<br>(In centimeters)
Weight	________________________________<br>(In kg, only digits, "dot" as decimal separator)
Up to what grade did you study?	◯ No education 1-4<br>◯ grade of incomplete elementary school 1-4 grade of<br>◯ complete elementary school 5-9 grade of incomplete<br>◯ elementary school 5-9 grade of complete elementary<br>◯ school<br>◯ Incomplete high school<br>◯ Completed high school<br>◯ Incomplete higher education<br>◯ Completed higher education

He has been arrested before	◯ Yes<br>◯ No
You work in prison	◯ Yes, currently<br>◯ No, but it has worked<br>◯ Never worked in prison
How many months have you worked or worked in prison?	______________________<br>(Full months or "1" if less than one month)
You smoke cigarettes (tobacco)	◯ Yes, currently<br>◯ No, but I have smoked<br>◯ Never smoked
Have you used any drugs in the last year?	☐ Did not use drugs<br>☐ Marihuana<br>☐ Cocaine<br>☐ Crack<br>☐ Heroin<br>☐ Glue and/or other solvents<br>☐ Base paste<br>☐ Hashish<br>☐ Injectable
Have you ever had tuberculosis?	◯ Yes<br>◯ No
You are treating tuberculosis	◯ Yes<br>◯ No
Have you ever had contact with someone with tuberculosis?	◯ I never had contact<br>◯ Yes, 1-3 times a week<br>◯ Yes, 4-6 times a week<br>◯ Yes, every day
You have the BCG vaccine mark on your right arm	◯ Yes<br>◯ No<br>(Check mark on arm)
There are people in your cell with coughs, fevers or losing weight.	◯ Yes<br>◯ No
Collector (sociodemographic data)	______________________

Machine Translated by Google

Symptoms

Record ID	______________________
Name: [common_arm_1][name]<br>Date of birth: [comum_arm_1][dt_nascimento]<br>TCLE signing date: [comum_arm_1][dt_tcle]	
Symptom screening date	______________________
Time for symptom screening	______________________
You have a cough	◯ Yes<br>◯ No
Cough for how many weeks	______________________<br>(Round up, should be > 0)
You have phlegm	◯ Yes<br>◯ No
Expectoration for how many weeks	______________________<br>(Round up, should be > 0)
Your sputum has blood in it	◯ Yes<br>◯ No
You have a fever	◯ Yes<br>◯ No
Fever for how many weeks	______________________<br>(Round up, should be > 0)
You have no appetite	◯ Yes<br>◯ No
Lack of appetite for how many weeks	______________________<br>(Round up, should be > 0)
You are losing weight	◯ Yes<br>◯ No
How many weeks have you been losing weight?	______________________<br>(Round up, should be > 0)

You have night sweats	◯ Yes<br>◯ No
Night sweats for how many weeks	______________________<br>(Round up, should be > 0)
You have chest pain	◯ Yes<br>◯ No
Chest pain for how many weeks	______________________<br>(Round up, should be > 0)
Are you having trouble breathing?	◯ Yes<br>◯ No
Difficulty breathing for how many weeks	______________________<br>(Round up, should be > 0)
Collector (symptoms)	______________________
